# Assessing the impacts of irrigation termination periods on cotton productivity under strategic deficit irrigation regimes

**DOI:** 10.1038/s41598-021-99472-w

**Published:** 2021-10-11

**Authors:** Sushil K. Himanshu, Srinivasulu Ale, James P. Bordovsky, JungJin Kim, Sayantan Samanta, Nina Omani, Edward M. Barnes

**Affiliations:** 1 Texas A&M AgriLife Research (Texas A&M University System), Vernon, TX 76385 USA; 2 Department of Food, Agriculture and Bioresources, Asian Institute of Technology, Khlong Luang, 12120 Thailand; 3 Texas A&M AgriLife Research (Texas A&M University System), Plainview, TX 79072 USA; 4 Institute of Environmental Technology, Seoul National University of Science and Technology, Seoul, 01811 South Korea; 5 Texas A&M University, College Station, TX 77843 USA; 6 Texas Community Watershed Partners, Texas A&M AgriLife Extension, Houston, TX 77058 USA; 7 Cotton Incorporated, Cary, NC 27513 USA

**Keywords:** Climate sciences, Hydrology

## Abstract

Determining optimum irrigation termination periods for cotton (*Gossypium hirsutum* L.) is crucial for efficient utilization and conservation of finite groundwater resources of the Ogallala Aquifer in the Texas High Plains (THP) region. The goal of this study was to suggest optimum irrigation termination periods for different Evapotranspiration (ET) replacement-based irrigation strategies to optimize cotton yield and irrigation water use efficiency (IWUE) using the CROPGRO-Cotton model. We re-evaluated a previously evaluated CROPGRO-Cotton model using updated yield and in-season physiological data from 2017 to 2019 growing seasons from an IWUE experiment at Halfway, TX. The re-evaluated model was then used to study the effects of combinations of irrigation termination periods (between August 15 and September 30) and deficit/excess irrigation strategies (55%-115% ET-replacement) under dry, normal and wet years using weather data from 1978 to 2019. The 85% ET-replacement strategy was found ideal for optimizing irrigation water use and cotton yield, and the optimum irrigation termination period for this strategy was found to be the first week of September during dry and normal years, and the last week of August during wet years. Irrigation termination periods suggested in this study are useful for optimizing cotton production and IWUE under different levels of irrigation water availability.

## Introduction

Irrigated agriculture in many semi-arid and arid regions across the globe has been threatened due to diminishing water resources. The semi-arid Texas High Plains (THP) is one of the most productive agricultural regions in the United States (US), mainly due to the availability of irrigation water from the underlying vast Ogallala Aquifer that is spread over eight states^1^. About 95% of the water pumped from this aquifer is used for irrigated agriculture^2^ and more than 90% of the region’s total water needs are met from this aquifer^4,5^. Producers in this region are finding it difficult to provide full crop water needs due to the rapid decline in groundwater levels and increased pumping costs^1,6–8^. The average decline in groundwater level between pre-development^9^ (~ 1950; the year after which extensive groundwater pumping for irrigation began) and 2015 was maximum in the Texas portion of the Ogallala Aquifer among eight states and it was estimated as 12.5 m^10^. The average decline across the entire Ogallala Aquifer region during the same period was estimated as 4.6 m^10^.

Cotton (*Gossypium hirsutum* L.) is a major irrigated crop grown in the THP region, contributing to about 25% of the US cotton production and 64% of the Texas cotton production^11,12^. Like other crops, cotton production in this region relies upon irrigation with water mined from the Ogallala Aquifer. Projected increase in temperature and more erratic precipitation patterns in the future could further intensify existing water shortages and adversely affect crop production in this region^13–15^. To prolong the Ogallala Aquifer’s life and sustain an agriculture-based economy, water districts in the THP regions have been restricting groundwater withdrawals from the aquifer. For example, the High Plains Water District (HPWD)^16^ and the North Plains Underground Water Conservation District^17^ have set an annual groundwater extraction limit at 460 mm (18 inches).

Rapidly declining groundwater levels and restrictions on the use of groundwater for irrigation necessitate the adoption of irrigation management strategies focused on water savings and economic enhancement. Even though cotton is highly adaptable to limited/deficit water conditions^18–20^, alternate approaches should be explored to reduce cotton irrigation demand. One such approach could be determining the optimum irrigation termination periods that can ensure efficient utilization and conservation of irrigation water while achieving higher crop yields. Irrigation termination is an important decision as cotton yield and fiber quality are affected by the timing and amount of rainfall and irrigation^20–25^. An early irrigation termination could result in a yield loss, while late termination may lead to undesirable vegetative growth, delayed boll maturity, increased pest management and harvest aid costs, as well as decreased irrigation productivity^20,26,27^. Identifying optimum irrigation termination periods for the THP region would, therefore, be important for efficient utilization of valuable irrigation water from the Ogallala Aquifer. Assessing the effect of irrigation termination dates on cotton irrigation water use efficiency (IWUE) and yield under different irrigation conditions (full/deficit) over multiple decades could result in more appropriate calendar-based recommendations for the THP region. Implementing appropriate irrigation termination strategies based on water availability could provide water savings with negligible/no yield losses.

Although several studies have investigated cotton responses to the timing and amount of irrigation, studies focusing on assessing the long-term impacts of irrigation termination timing on cotton production are limited. Based on field experiments, contrasting recommendations were made on the timing of irrigation termination for cotton without losing yield and fiber quality (Table 1). These field experiments were carried out under varying soil and climatic conditions and different irrigation systems. Several researchers reported that extending irrigation too late into the season can reduce productivity due to excessive vegetative development and cause lodging of plants, increase difficulty in defoliation, and increase boll rotting^28–31^. No gain in yield or fiber quality was observed with irrigation after bolls begin to open. Spray irrigation following boll opening provided moisture in the crop canopy which enhanced the environment for the plant pathogens that cause boll rot or hardlock^31^. As compared to pivot irrigated fields, irrigation termination in furrow irrigated fields was recommended a few days before^29–31^. Some researchers suggested irrigation termination periods based on growing degree days (GDD) after physiological cutout and their recommendations varied substantially^27,29,32,33^. They have considered the appearance of five nodes above white flower on the main stem as the physiological cutout stage. Lascano et al.^24^ evaluated irrigation termination thermal times based on accumulated heat units from crop emergence (Table 1). Masasi et al.^34^ emphasized that irrigation termination decisions should be made based on the amount and timing of late-season precipitation events. Soil water holding capacity^28,35^, weather conditions^36–38^ and maturity timings for cotton varieties^39^ also affected irrigation termination decisions. The differing results from these studies illustrate that the optimal irrigation termination time for cotton can vary by region and management, necessitating a further investigation of the long-term impacts of irrigation termination on cotton yield under different weather conditions.

**Table 1:** Previous recommendations on irrigation termination periods for cotton based on different field experiments.

[Reference] Location and experiment period	Climatic condition	Soil types	Irrigation system	Planting period	Results/Recommendation
^24^ Texas A & M AgriLife Research, Lubbock, Texas (1996–1999)	Semi-arid	Olton clay loam	Low energy precision application	Early May to end of May	Irrigation termination suggested at cumulative daily heat units (from crop emergence) of 890 °C in high (7.6 mm d^-1^), and 1000 °C in low (2.5 mm d^-1^) and medium (5.1 mm d^-1^) irrigation level treatments
^27^ 28 cotton fields in states of Arkansas, Louisiana, Mississippi, Missouri, and Texas (2000–2007)	Sub-tropical	Silty loam; Sandy loam; Clay; Silty clay	Furrow irrigation	Mid-April to end of May	Optimal irrigation termination recommended at 192 GDD after physiological cutout
^28^ San Joaquin Valley, California (1954–1968)	Semi-arid	Hesperia fine sandy loam; Panoche clay loam	Furrow irrigation	Early April (early May in 1967)	Final irrigation should be given much earlier on a high water-retaining soil (Panoche clay loam) than on a low water-retaining soil (Hesperia fine sandy loam)
^29^ 27 furrow-irrigated and 43 pivot-irrigated fields, Arkansas (2005–2012)	Sub-tropical	Heterogenous soils	Furrow and pivot irrigation	End of April to early May	Optimal irrigation termination recommended at 350 GDD after physiological cutout. Irrigation termination recommended approximately 8 days before in furrow irrigated fields as compared to pivot irrigated fields
^32^ 19 cotton fields in states of Arkansas, Louisiana, Mississippi, and Missouri (2000–2004)	Sub-tropical	Silty loam; Sandy loam; Silty clay	Furrow irrigation	Mid-April to end of May	Optimal irrigation termination recommended at 336 GDD after physiological cutout
^33^ St. Lawrence, Texas (2003)	Semi-arid	Not reported	Sub-surface drip irrigation	Mid May	Optimal irrigation termination recommended at 300—400 Growing Degree Days (GDD) after physiological cutout
^34^ Oklahoma State University, Oklahoma (2015–2017)	Sub-humid	Hollister silty clay loam	Open canal	Late May to early June	Increase in cotton yield reported when irrigation termination periods moved from mid- to end- August
^35^ University of Arizona, Tucson, Arizona (2000–2002)	Arid/semi-arid	Casa Grande sandy loam; Indio clay loam	Furrow irrigation	End of March to mid-April	Significant increase in yield reported with later (late September) irrigation termination
^36^ Southern High Plains, Texas (1984–1987)	Semi-arid	Sandy loam	Sprinkler irrigation	Mid May to early June	Terminating irrigation at first open boll limited vegetative growth and led to higher yields
^37^ Punjab Agricultural University, India (2000–2003)	Tropical, Semi-arid	Sandy loam	Surface flooding	Early April to early May	A significant increase in cotton yield was reported with later irrigation termination
^38^ Texas Tech University, Lubbock, Texas (2010–2011)	Semi-arid	Not reported	Sub-surface drip irrigation	Early to late May	Early irrigation termination was found desirable in a dry year for saving water for future use
^39^ Bekaa Valley, Lebanon (2001–2002)	Mediterranean	Clay	Drip irrigation	Early to mid-May	Terminating irrigation at first open boll resulted in higher cotton yield as compared to later irrigation termination

Evaluating efficient irrigation strategies through field experiments alone is rather difficult as late-season rainfall events can lead to the loss of an experimental site-year and results need to be accumulated over different weather scenarios (hot and dry, cold and wet, etc.). Crop growth models, on the other hand, are very useful to rapidly simulate different crop growing conditions and assess their long-term impacts on crop production and enable the development of efficient irrigation strategies for different agro-climatic conditions^19,40,41^. However, these models require a rigorous calibration and evaluation against measured data before using them for strategy development. Several crop simulation models are available and are capable of effectively simulating cotton growth and development under various management schemes^40^. The CROPGRO-Cotton module within the Decision Support System for Agrotechnology Transfer (DSSAT) Cropping System Model (CSM) can serve as an effective tool for simulating cotton growth and yield under varied soil, crop management and weather conditions^40,42,43^. The DSSAT CSM CROPGRO-Cotton model has been used extensively by researchers worldwide for suggesting efficient irrigation management strategies for cotton production^19,20,37,44–49^.

Limited studies have used crop growth models for determining optimum irrigation termination periods for cotton^20^. In our previous study^20^, we suggested ideal irrigation termination periods for the THP using the DSSAT CSM CROPGRO-Cotton model, which was calibrated by Adhikari et al.^50^ using measured data from 2010 to 2013 growing seasons from an IWUE experiment conducted by Bordovsky et al.^51^ at the Texas A&M AgriLife Research Center at Halfway in the geographic center of the THP. However, those irrigation termination recommendations were given for a full and three deficit irrigation treatments implemented by Bordovsky et al.^51^ and such recommendations for commonly adopted ET-replacement-based deficit irrigation strategies are lacking. The IWUE experiments were resumed at Halfway in 2016 with additional late-season irrigation treatments, and detailed in-season crop physiological data was collected from this experiment during the 2017—2019 growing seasons. Re-evaluating the CROPGRO-Cotton model based on this updated yield dataset, which includes late-season irrigation termination treatments, should enhance confidence in the use of the model for determining optimum irrigation termination dates for commonly adopted ET-replacement based irrigation strategies. The objectives of this study were therefore to: (1) re-evaluate the Adhikari et al.^50^ calibrated CROPGRO-Cotton model using additional in-season crop physiological and yield data available from 2017 to 2019 field experiments conducted at Halfway, TX, (2) assess the long-term (1978–2019) effects of irrigation termination periods on seed cotton yield and IWUE under commonly adopted deficit/excess ET-replacement practices, and (3) suggest the optimum irrigation termination periods for cotton production under strategic deficit/excess irrigation regimes during dry, normal, and wet years.

## Results and discussion

### Model evaluation

Parameters adjusted during the DSSAT-CSM-CROPGRO-Cotton model calibration are presented in Supplementary Table S1. Initial soil water content was also adjusted during the calibration and the calibrated initial soil water for years 2017, 2018 and 2019 was 0.10, 0.21 and 0.16 cm^3^ cm^−3^, respectively. Results from calibration of the model for simulation of cotton phenological stages, canopy height and seed cotton yield are presented in the following sections.

### Cotton phenological stages

Simulated dates of onset of different cotton phenological stages were within the range of measured dates during both calibration and evaluation of the model (Supplementary Table S2). The measured dates of onset of phenological stages were highly variable during the field experiment due to the differences in irrigation amounts applied under different treatments and variability in growing season temperature during the years of field experiment. The anthesis and physiological maturity stages occurred earlier during the year 2018 as compared to other years due to higher air temperatures (seasonal average maximum temperature was 28.0 °C, 29.1 °C and 28.3 °C during the years 2017, 2018 and 2019, respectively), which resulted in rapid growth and the shorter time interval between developmental stages. Similarly, physiological maturity was delayed in the year 2017 due to relatively lower temperatures during later periods of the growing season. The cultivar parameter EM-FL (time between plant emergence and flower appearance) and the ecotype parameters PL-EM (time between planting and emergence) and EM-V1 (time required from emergence to first true leaf) were adjusted during calibration to obtain a reasonable match between the simulated and measured dates of onset of growth stages (Supplementary Table S1).

### Cotton canopy height

Measured and simulated canopy heights in different irrigation treatments matched well during the model calibration (Supplementary Figure S1) and evaluation (Supplementary Figure S2). Simulated canopy height was within the range of measured heights, except in a few cases during the year 2018. The simulated canopy height was, in general, overpredicted during the calibration (high irrigation treatments) and underpredicted during the evaluation (deficit irrigation treatments) in all years. Modala et al.^45^ also reported underprediction of canopy height in 75% ET-replacement treatment. The use of air temperature, instead of canopy temperature for crop growth simulation, could be a reason for the underprediction/overprediction of canopy height^40^. The model ecotype parameters RWDTH (relative width of the ecotype in comparison to the standard width per node), TRIFL (rate of appearance of leaves on the mainstem, leaves per photothermal day) and RHGHT (relative height of the ecotype in comparison to the standard height per node) were found to affect canopy height simulation and hence they were calibrated to values of 1.0, 0.18 and 0.80, respectively (Supplementary Table S1). Overall, the model performance statistics in canopy height prediction in this study were very good (Supplementary Table S3) and they were comparable to values achieved in previous studies^45,49^.

### Seed cotton yield

**Figure 1:**
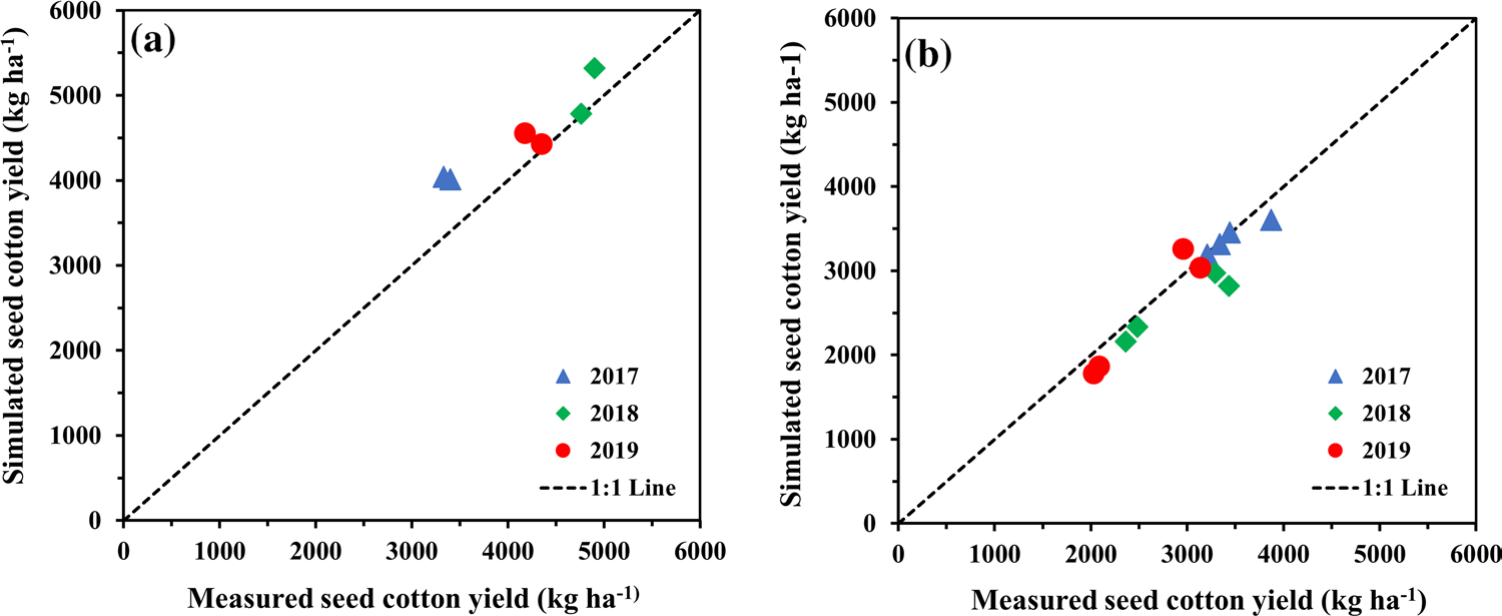
Comparison of measured and simulated seed cotton yield during model: (**a**) calibration, and (**b**) evaluation over 2017–2019 growing seasons.

The measured and simulated seed cotton yields were in good agreement during the model calibration and evaluation (Fig. 1, Table 2). Model performance during the evaluation was comparable to that of Adhikari et al.^50^, and the evaluated model in this study has nicely captured the effects of differences in late season irrigation on seed cotton yield. The model performance in seed cotton yield prediction was slightly better during the calibration (high irrigation treatments) than the evaluation (water limiting treatments) (Table 2). These results reinforce outcomes from earlier studies that reported significant underprediction of seed cotton yield under drier conditions^40,45,52^.

**Table 2:** Model performance statistics for seed cotton yield simulation.

Model performance statistics	Calibration	Evaluation
Index of agreement (d-index)	0.96	0.95
Coefficient of determination (r^2^)	0.93	0.88
Percent root mean square error (RMSE)	5.47	8.83
Average percent error (PE)	3.66	− 5.18

### Impact of irrigation termination date on simulated seed cotton yield and irrigation water use

**Figure 2:**
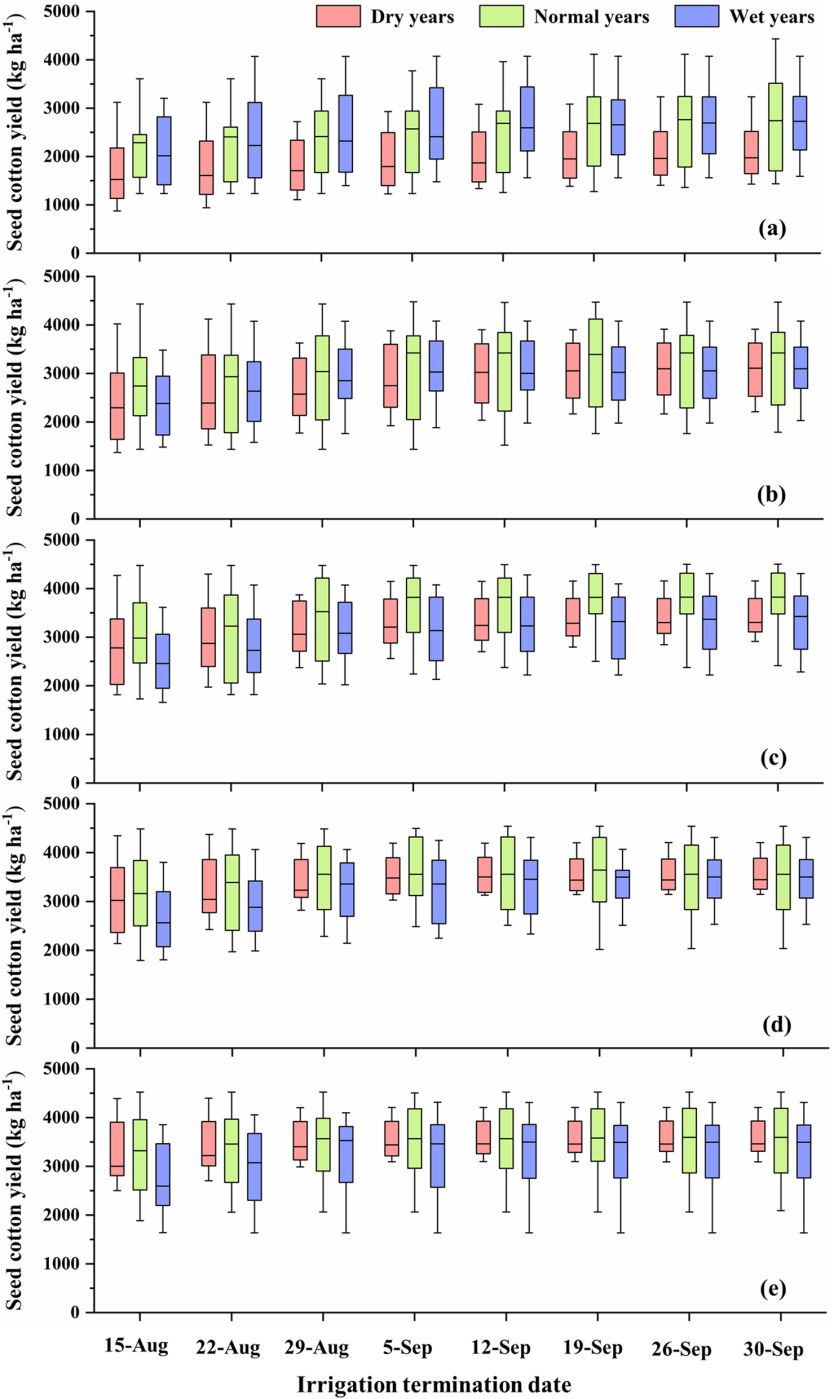
Effect of irrigation termination date on simulated seed cotton yield under: (**a**) 55%, (**b**) 70%, (**c**) 85%, (**d**) 100%, and (**e**) 115% ET-replacement strategies. The ends of the boxes indicate 25th and 75th percentiles, and the horizontal line inside the box indicates the median.

**Figure 3:**
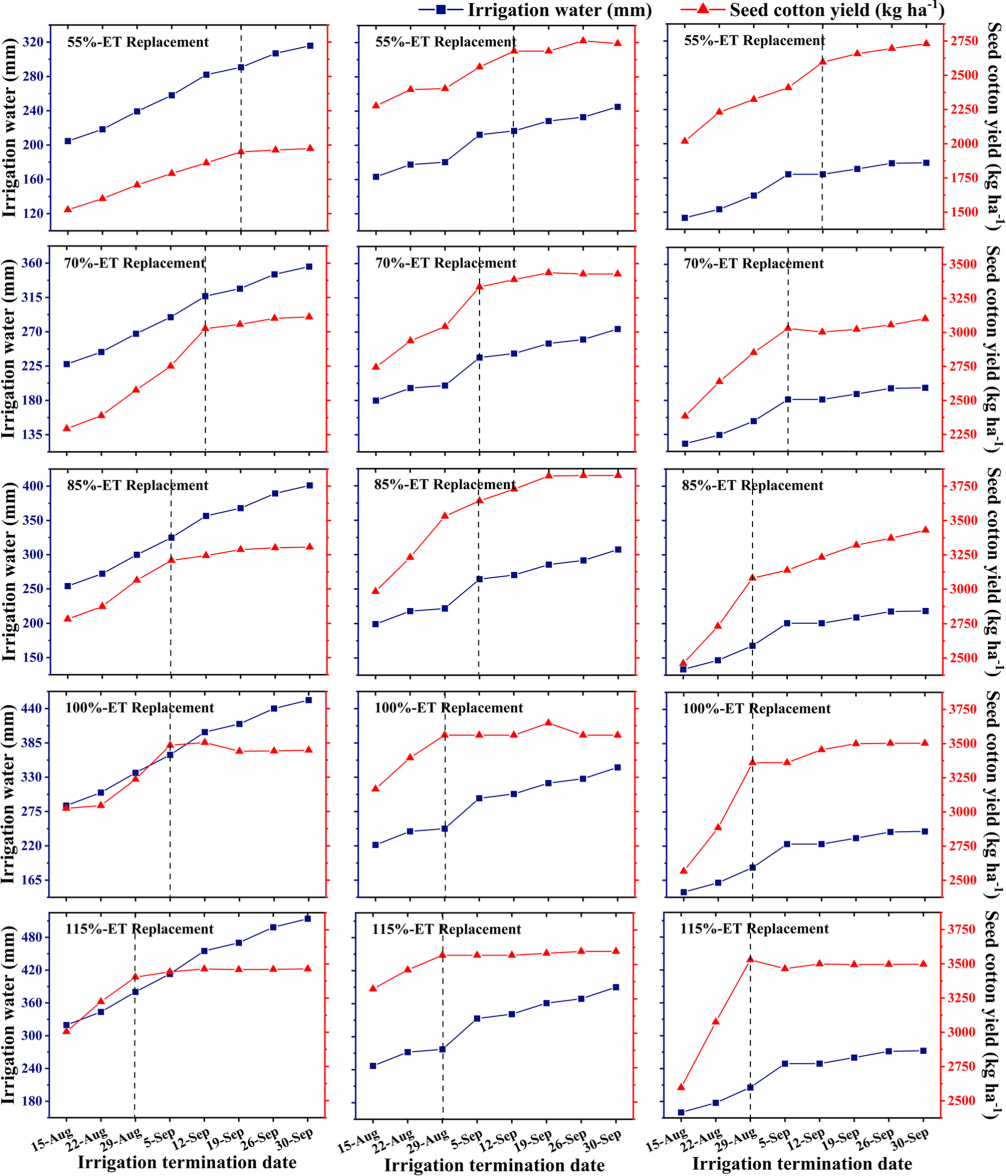
Effect of irrigation termination date on median irrigation water use and seed cotton yield under different excess/deficit irrigation strategies. The vertical dotted line indicates the irrigation termination date after which an increase in average seed cotton yield from that termination date to the next termination date was < 3%. (Left, center and right panels correspond to dry, normal, and wet years, respectively).

In general, as the irrigation termination date moved towards the end of the growing season, simulated seed cotton yield increased rapidly until a certain termination date, and then the rate of increase has either declined substantially or became negligible for the remaining termination dates (Fig. 2). Percent changes in median seed cotton yield from a termination date to the next termination date were then calculated, and ideal irrigation termination dates were identified based on our selected criteria (the earliest termination date at which the simulated median seed cotton yield reached a peak value or when an increase in median seed cotton yield from that termination date to the next termination date was < 3%) (Fig. 3).

As expected, the ideal irrigation termination period occurred later in the season in case of deficit irrigation strategies (55%, 70% and 85% ET-replacement) when compared to full/excess irrigation strategies (100% and 115% ET-replacement) (Fig. 3). Based on the simulated median seed cotton yield, the ideal irrigation termination dates under 55%, 70%, 85%, 100% and 115% ET-replacement strategies were identified as September 12, September 5, September 5, August 29 and August 29, respectively under normal weather conditions. In the case of wet years, ideal irrigation termination periods were the same as those under normal years, except for the 85% ET-replacement strategy when the ideal termination period was found to be a week earlier. Ideally, we expect irrigation termination periods in wet years to be one/two weeks earlier when compared to normal years. However, interestingly, the average precipitation received during mid-August to mid-September in wet years (~ 56 mm) was less than that received in normal years (~ 60 mm), which ultimately affected the ideal irrigation termination dates. In the case of dry years, ideal irrigation termination periods were found to be a week later than those in normal years for 55%, 70% and 100% ET replacement strategies, and similar to those in normal years for 85% and 115% ET-replacement strategies. The inconsistency in ideal irrigation termination date in case of 100% ET replacement strategy was due to 57 mm of precipitation received between August 29 and September 5 in the dry year 2002, which resulted in a huge yield gain (much higher than the 3% threshold used in our selection criteria) under 100% ET-replacement strategy, and shifted the ideal termination date by a week. A few other inconsistencies in the trends of simulated median seed cotton yield were also due to reclassification of dry, normal and wet years for each termination date based on the growing season precipitation received until that termination date, which resulted in some years being classified under different categories for different termination dates (e.g., year 1995 was classified as a normal year until the termination date of September 12 and as a wet year for later termination dates).

The simulated seed cotton yield was found to be the lowest under 55% ET-replacement strategy followed by the 70% ET-replacement strategy, as expected (Fig. 2a,b). The 85% ET-replacement irrigation strategy was found to be beneficial for conserving irrigation water as it resulted in no/slight decrease in seed cotton yield as compared to the 100% ET-replacement strategy (Fig. 2c,d). Providing excess irrigation than the ET requirement (115% ET-replacement) did not contribute to any increase in simulated seed cotton yield (Fig. 2d,e). In wet years, excess irrigation than the ET requirement (115% ET-replacement) has even resulted in a decrease in seed cotton yield due to excess water stress. Interestingly, simulated seed cotton yield was found to be the highest in normal years under most irrigation strategies. In some cases (e.g., irrigation termination dates of August 15 and August 22 in the case of 85%, 100% and 115% ET-replacement strategies), simulated seed cotton yield in wet years was lower than that in dry years. This was also due to excess water stress caused by heavy rains received during the beginning of the growing season. Similar findings were reported by other researchers who noted that humid and warm climate is favorable for cotton^19,53–55^, and wet weather conditions for prolonged duration could lead to yield loss^56–58^. In general, simulated seed cotton yield under deficit irrigation strategies (55%, 70% and 85% ET-replacement) was found to be higher under normal/wet years as compared to dry years. However, in the case of full/excess irrigation strategies (100% and 115% ET-replacement), no significant differences in simulated seed cotton yield were found between dry and normal/wet years, especially when irrigation was terminated at later dates.

The simulated median irrigation water use increased continuously as the irrigation termination date moved towards the end of the growing season (Fig. 3). As expected, simulated median irrigation water use was the highest in dry years for all irrigation strategies followed by normal and wet years (Fig. 3). Simulated median irrigation water use was less than the annual groundwater pumping limit (460 mm) specified by the HPWD under all irrigation scenarios among all categories of years, except for the 115% ET-replacement strategy during dry years under irrigation termination dates of September 26 and September 30 (Fig. 3). Ale et al.^20^ also reported that cotton irrigation water use under full irrigation may exceed the HPWD’s pumping limit if irrigation is terminated after 5 September in dry years and after 12 September in normal and wet years. Overall, these results indicate that adoption of appropriate deficit irrigation strategies along with suggested termination dates could enable producers achieve higher IWUE with minimum/no reduction in seed cotton yield while being compliant with the HPWD regulations.

### Impact of irrigation termination date on simulated irrigation water use efficiency (IWUE)

**Figure 4:**
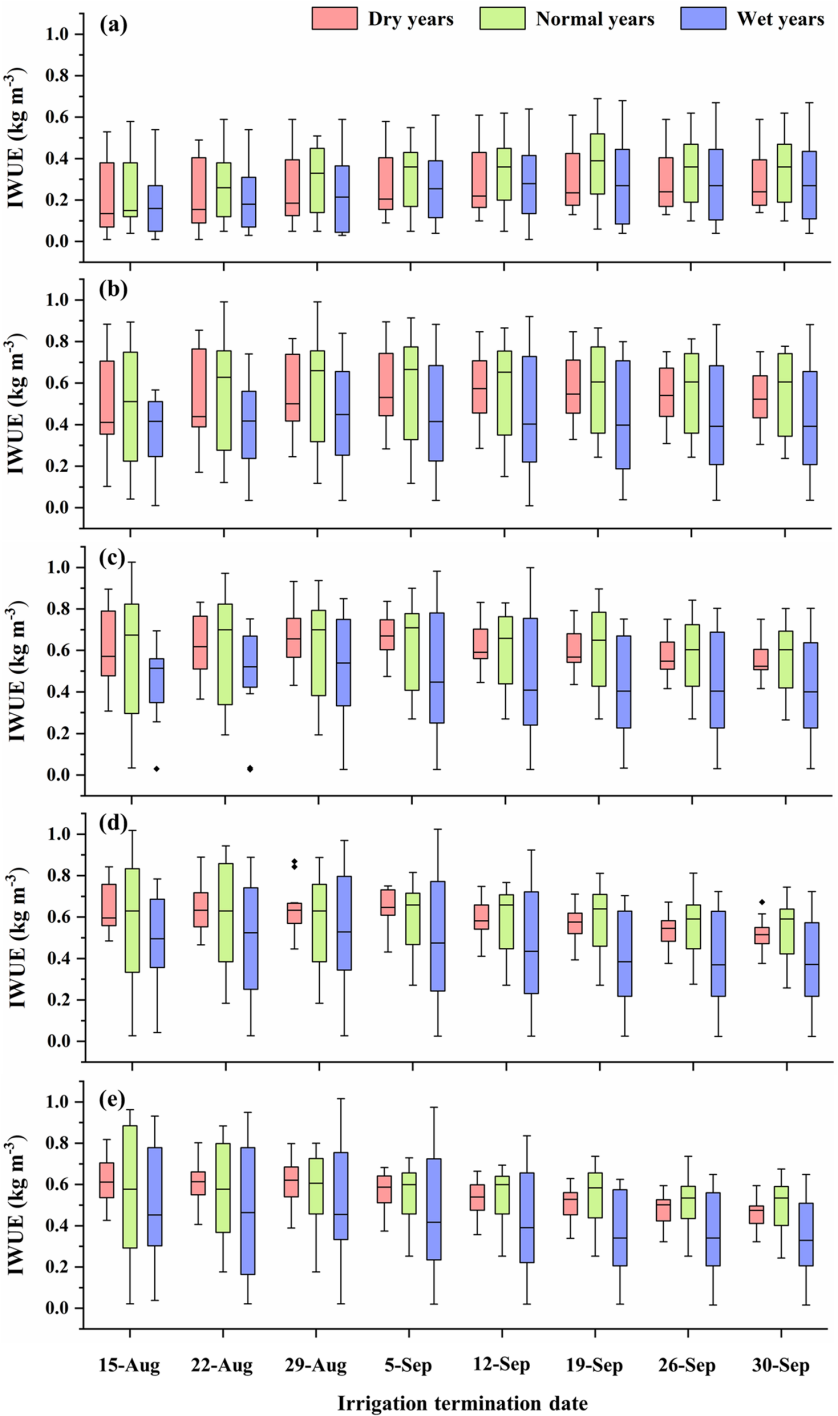
Effect of irrigation termination date on simulated irrigation water use efficiency (IWUE) under: (**a**) 55%, (**b**) 70%, (**c**) 85%, (**d**) 100% and (**e**) 115% ET-replacement strategies. The ends of the boxes indicate 25th and 75th percentiles, and the horizontal line inside the box indicates the median.

In general, as the irrigation termination date moved towards the end of the growing season, the median simulated IWUE increased until a certain termination date and then decreased for the later termination dates. Ideal irrigation termination dates were identified based on our selected IWUE criteria (date on which simulated median IWUE was the highest or the earliest irrigation termination date in the case of same maximum IWUE on multiple dates). Based on the simulated median IWUE, the ideal irrigation termination dates in normal years were identified as September 19, September 5, September 5, September 5 and August 29 under 55%, 70%, 85%, 100% and 115% ET-replacement strategies, respectively (Fig. 4). In the case of wet years, ideal irrigation termination periods were found to be a week earlier than those in normal years under all irrigation strategies. In the case of dry years, ideal irrigation termination periods were the same as those under normal years, except for the 70% ET-replacement strategy when the ideal termination period was found to be a week later (Fig. 4). Ideally, we expect irrigation termination periods in dry years to be one/two weeks later when compared to normal years. However, as discussed earlier, a substantial amount of precipitation (57 mm) was received between August 29 and September 5 in the dry year 2002, which resulted in a huge yield gain and it ultimately affected the ideal irrigation termination periods for different ET-replacement strategies under dry years.

As expected, simulated median IWUEs were lower in wet years under all irrigation strategies, except for the 55% ET-replacement strategy in which simulated IWUE was higher in wet years as compared to dry years (Fig. 4). The simulated IWUEs were higher in normal years under most irrigation strategies, except the 115% ET-replacement strategy with earlier termination dates when simulated IWUEs were higher in dry years. Higher IWUEs in normal years as compared to wet years under most irrigation strategies further emphasize that the prolonged wet weather conditions are not favorable for cotton production^55–58^.

Simulated IWUE was the highest for 85% ET replacement strategy and the lowest for 55% ET replacement strategy among the most termination dates and categories of years (Fig. 4). Other studies from this region have also found the 85% ET replacement to be a promising deficit irrigation strategy for cotton production^45,59^. Interestingly, simulated IWUE in the case of 115% ET-replacement was lower than that of 100% and 85% ET-replacement strategies in all weather conditions. A similar trend was also reported in other studies^45,60^. The THP region receives an average annual rainfall of 470 mm, out of which a major portion (about 330 mm) occurs during the cotton growing season^51^, and this rainfall is generally adequate to achieve a good yield of a drought-tolerant crop such as cotton under deficit irrigation strategies. Modala et al.^50^ also reported a marginal reduction in yield in the adjacent Texas Rolling Plains region when irrigation was decreased from 100 to 66% ET-replacement under normal rainfall conditions; however, the reduction was substantial under dry conditions. On the other hand, due to semi-arid and windy climate along with uncertain rainfall patterns, maintaining high IWUE is a challenge in the THP region^51^, and irrigation as per lower ET-replacement (e.g., 55% ET-replacement) strategies could lead to substantial yield loss and reduction in IWUE.

### Suggested optimum irrigation termination periods under different excess/deficit irrigation strategies

**Figure 5:**
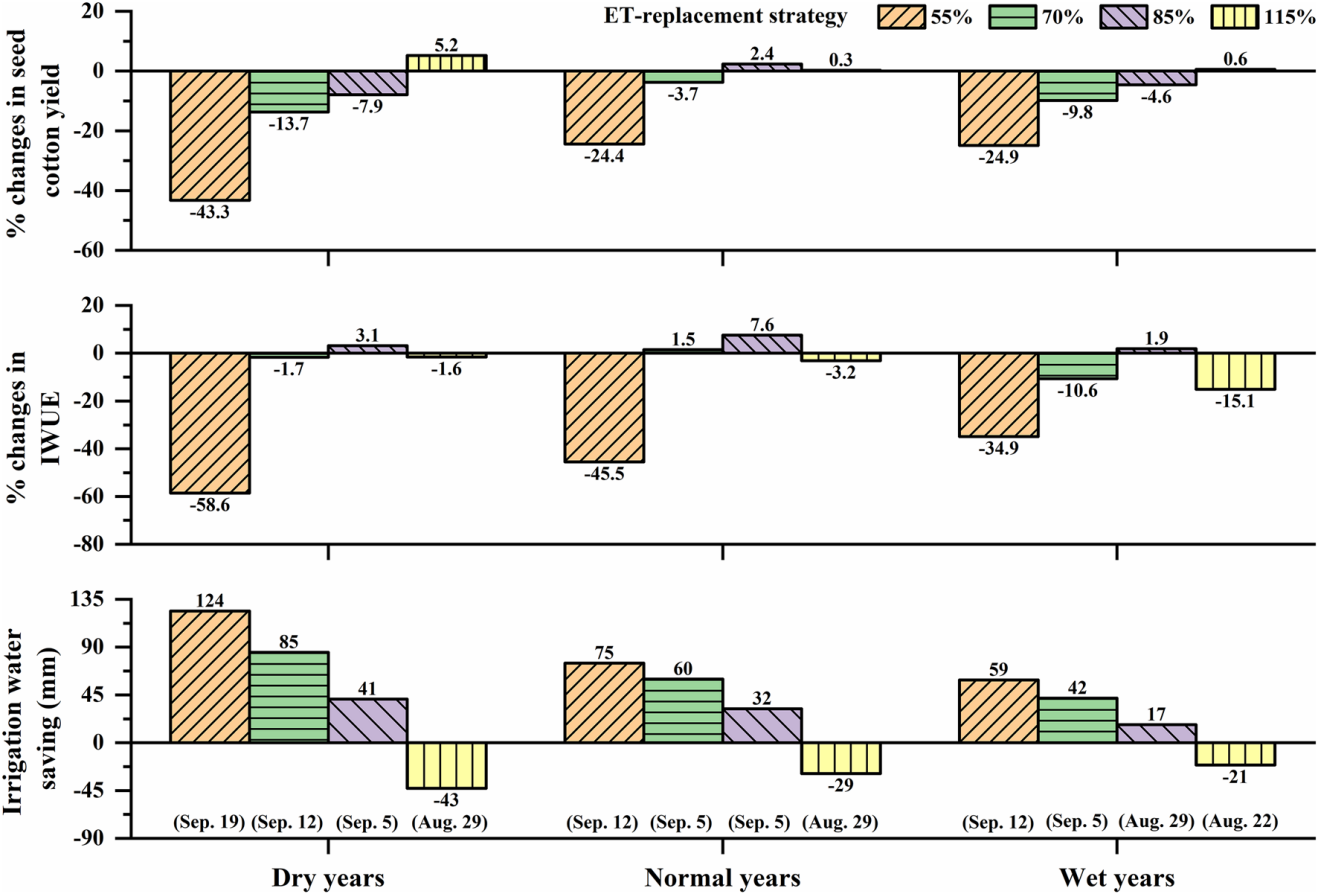
Effects of irrigation termination periods on percent changes [increase (+)/decrease (−)] in seed cotton yield (top panel) and IWUE (center panel), and differences in the amount of irrigation water (bottom panel) under different ET-based irrigation strategies (55%, 70%, 85% and 115% ET replacement) as compared to 100% ET-replacement irrigation strategies. The suggested irrigation termination periods under different ET-based irrigation strategies are shown in parentheses at the bottom of respective bars.

Ideal irrigation termination periods identified for different excess/deficit irrigation strategies based on median seed cotton yield and IWUE were mostly similar, except for 100% and 55% ET-replacement strategies in normal years, and 115% and 70% ET-replacement strategies in wet years (Table 3). In normal years, there was an insignificant increase in yield when irrigation termination date was changed from August 29 to September 5 in the case of 100% ET-replacement strategy (*P*-value = 0.39), and from September 12 to September 19 in the case of 55% ET-replacement strategy (*P*-value = 0.62) (Fig. 5). Therefore, August 29 and September 12 were considered as the optimum irrigation termination periods for 100% and 55% ET-replacement strategies, respectively in normal years as higher IWUE can be achieved with these termination dates with negligible yield loss. In contrast, in wet years, increase in yield was significant when irrigation termination date was changed from August 22 to August 29 in the case of 115% ET-replacement strategy (10% increase), and from August 29 to September 5 in the case of 70% ET-replacement strategy (6.5% increase) (Fig. 5). In addition, simulated irrigation water use was substantially lower than the annual groundwater extraction limit when irrigation was terminated on August 29 (206 mm) in case of 115% ET-replacement strategy and on September 5 (181 mm) in case of 70% ET-replacement strategy. August 29 and September 5 were therefore considered as the optimum irrigation termination periods for 115% and 70% ET-replacement strategies, respectively, in wet years (Table 3).

**Table 3:** Suggested optimum timings of irrigation termination for excess/deficit irrigation treatments in dry, normal and wet years.

Climate category	ET-Replacement strategy^a^	Ideal irrigation termination date based on simulated median seed cotton yield^b^	Ideal irrigation termination date based on simulated median IWUE^c^	Suggested optimum irrigation termination period^d^	Irrigation water saving/loss (mm)^e^
Dry years	55%	September 19 (1951)	September 19 (0.24)	September 19 (132)	124
	70%	September 12 (3023)	September 12 (0.57)	September 12 (125)	85
	85%	September 5 (3208)	September 5 (0.67)	September 5 (118)	41
	100%	September 5 (3483)	September 5 (0.65)	September 5 (118)	0
	115%	August 29 (3404)	August 29 (0.62)	August 29 (111)	− 43
Normal years	55%*	September 12 (2688)	September 19 (0.39)	September 12 (125)	75
	70%	September 5 (3329)	September 5 (0.67)	September 5 (118)	60
	85%	September 5 (3641)	September 5 (0.71)	September 5 (118)	32
	100%*	August 29 (3557)	September 5 (0.66)	August 29 (111)	0
	115%	August 29 (3567)	August 29 (0.61)	August 29 (111)	− 29
Wet years	55%	September 12 (2596)	September 12 (0.28)	September 12 (125)	59
	70%*	September 5 (3030)	August 29 (0.45)	September 5 (118)	42
	85%	August 29 (3205)	August 29 (0.54)	August 29 (111)	17
	100%	August 29 (3360)	August 29 (0.53)	August 29 (111)	0
	115%*	August 29 (3379)	August 22 (0.46)	August 29 (111)	− 21

^a^Strategies indicated by a star symbol had differences in ideal termination periods identified based on median seed cotton yield and IWUE.

^b^Values in parentheses are median seed cotton yields in kg ha^-1^.

^c^Values in parentheses are median IWUEs in kg m^-3^.

^d^Values in parentheses are days after planting, DAP.

^e^Saving (+)/loss (−) in irrigation water under suggested optimum irrigation termination period as compared to irrigation water used in 100% ET replacement strategy.

The suggested optimum irrigation termination periods for full/excess irrigation strategies (100% and 115% ET-replacement) varied from the last week of August to the first week of September while those for the deficit irrigation strategies (55%, 70% and 85% ET-replacement) varied from the first week of September to the third week of September. The optimum irrigation termination periods suggested in this study are either the same or one week earlier than those suggested by Ale et al.^20^ for the THP region. This was mainly due to the application of pre-plant irrigation in all strategies simulated in this study. Pre-plant irrigation enables establishment of a more effective root zone early in the growing season^61,62^. Depending on soil water content at the time of planting and availability of irrigation water, producers in the THP region also generally apply pre-plant and/or at-plant irrigations to establish a good plant stand^51,61^. The optimum irrigation termination periods suggested in this study are comparable to prior studies^24,29,34,39^.

In comparison to 100% ET-replacement strategy, simulation of 115% ET-replacement strategy (with 43 mm, 29 mm and 21 mm of excess irrigation water in dry, normal and wet years, respectively) resulted in only a small improvement in yield (5.2%, 0.3% and 0.6% increase in dry, normal and wet years, respectively), but caused a substantial decline in IWUE (1.6%, 3.2% and 15.1% decline in dry, normal and wet years, respectively) (Fig. 5). Cotton does not adapt well to excess water stress in the root zone, and prolonged periods of excess water stress can significantly reduce the yield^63,64^. Simulated seed cotton yield and IWUE were found to be the lowest in the case of 55% ET-replacement strategy in all categories of years (Fig. 5). Overall, the 85% ET-replacement irrigation strategy was found to be the most efficient strategy in terms of saving irrigation water. The 85% ET-replacement irrigation strategy resulted in maximum IWUE in all weather conditions. However, the simulated median seed cotton yield decreased by 7.9% and 4.6% in dry and wet years, respectively, with the 85% ET-replacement strategy as compared to the 100% ET-replacement strategy (Fig. 5). Interestingly, a slight increase (2.4%) in median seed cotton yield was found in normal years under the 85% ET-replacement strategy. By adopting the 85% ET-replacement strategy along with the suggested optimum irrigation termination dates (September 5, September 5 and August 29 in dry, normal and wet years, respectively), about 41, 32 and 17 mm of irrigation water could be saved annually (compared to the 100% ET replacement strategy). These results indicate that strategic implementation of deficit irrigation strategies along with appropriate dates of irrigation termination could increase IWUE and ensure good cotton yield^20,59^. However, the recommendations from this study should be used with caution, as the irrigation termination decisions for cotton depend on different variables including expected yield and fiber quality, market value and irrigation costs^24^. Several other factors such as soil type, soil water status, irrigation system used, growth stage, geographic location and crop health^29–31^ and maturity timings for cotton varieties^65^ could also affect the irrigation termination decisions. As the growing season progresses, depending on the actual amount of precipitation received and based on short-term forecasted weather data, changes to irrigation termination decisions may be necessary. In addition, crop water productivity (CWP)^66^, which considers the contribution from rainfall and soil water content (in addition to irrigation) to crop yield, could serve as a better indicator for determining optimum irrigation periods than IWUE and our future efforts will focus on this improvement.

## Methods

### Cotton IWUE experiment at halfway

A cotton IWUE experiment was conducted at the Texas A&M AgriLife Research Center at Halfway (34° 10ʹ N, 101° 56ʹ W; elevation 1075 m) (Supplementary Figure S4) during the 2010–2013 growing seasons and measured data from this experiment was previously used to evaluate the CROPGRO-Cotton model^50^. That field experiment consisted of 27 treatments with three different maximum irrigation rates (0 mm d^−1^–Low (L), 3.2 mm d^−1^–Medium (M), and 6.4 mm d^−1^–High (H)) implemented in combination with three cotton growth stages (vegetative, reproductive and maturation)^49^. These three growth stages were defined based on accumulated growing degree days at a threshold temperature of 15.6 °C^51^. Treatments were labeled by the irrigation water level during each growth stage (for example, LHH treatment received Low irrigation rate during vegetative growth, and High irrigation rate during reproductive and maturation growth stages). A low energy precision application (LEPA) center pivot irrigation system was used for irrigation applications. More details about the field experiment and previous model evaluation can be found in Bordovsky et al.^51^ and Adhikari et al.^50^, respectively. Following a two-year period (2014 and 2015) used to equalize soil water and residual nutrient content across plots, IWUE experiments were resumed at the same location as the 2010–2013 experiments in 2016 by modifying treatments to include late, as well as more typical irrigation termination treatments. We collected in-season crop physiological data (dates of onset of cotton phenological stages and canopy height) and end-of-the-season yield data from this new experiment during the 2017–2019 growing seasons and we re-evaluated the Adhikari et al.^50^ CROPGRO-Cotton model based on this new data as a part of this study. Irrigation treatments considered for model re-evaluation included LMM, LMH, HHH, LMM + , LMH + , and HHH + (+ symbol indicates late irrigation termination). The soil at the study site is Pullman clay loam (fine, mixed, super active, thermic Torrertic Paleustolls). The average (1978–2019) growing season (April–October) and annual rainfall at the study site were 376 mm and 463 mm, respectively. The recorded minimum and maximum temperatures were − 21.3 °C and 44.4 °C, respectively.

### The crop simulation model used and the model inputs

The DSSAT CSM CROPGRO-Cotton model (version 4.7) was used in this study. The model uses weather data, genetic parameters, and soil and crop management data as inputs to simulate crop growth and development over time in response to different crop and soil management practices^40,42,67,68^. The DSSAT comprises crop simulation models for over 42 crops (as of Version 4.7.5) as well as different tools to facilitate the effective use of the models^42,67^. The model calculates and reports model state variables over daily time steps^69^. Long-term (1978–2019) daily weather data for this study were obtained from an on-site weather station. Daily weather data input to the model included precipitation, maximum and minimum air temperature, solar radiation, dew point temperature, relative humidity and wind speed. The missing weather data were filled with the data retrieved from nearby Olton 6S and Plainview 1S weather stations, which are part of the West Texas Mesonet^70^. A summary of monthly average weather parameters during the model re-evaluation period of 2017–2019 cotton growing seasons (April through October) is presented in Supplementary Table S4. Required genetic parameter inputs include ecotype, cultivar and species coefficients defined in ECO, CUL, and SPE suffixed files, respectively. Tillage, planting, harvesting, fertilizer/chemical application related information was input according to the actual practices adopted in the field experiment (Supplementary Table S5). The details about the soil input parameters can be found in Adhikari et al.^50^.

### Model evaluation with the new in-season physiological data and yield data

The CROPGRO-Cotton model was re-evaluated using the most recent in-season crop phenological data and cotton yield data collected during 2017–2019. The model was calibrated based on measured data from two high irrigation treatments (HHH and HHH +) and evaluated against measured data from four deficit irrigation treatments (LMM, LMH, LMM + and LMH +). A new cultivar FiberMax 2011GT was added to the DSSAT cultivar database. The cultivar and ecotype parameters for this variety were populated based on the literature values^71^ and the calibrated values reported for the THP region in previous studies^50,52^. The testing ranges used in Adhikari et al.^50^ were slightly modified in this study to account for the differences in cultivars. Some of the sensitive cultivar and ecotype parameters were then adjusted until a good match between the measured and simulated values was achieved. The parameters related to crop growth were initially adjusted to achieve a good match between the simulated dates of onset of emergence, anthesis and physiological maturity stages, and the measured dates from the field experiment. The parameters related to crop development and yield were then adjusted until a good agreement between the simulated and measured seed cotton yield was achieved. A similar approach of calibrating phenological stages before calibrating parameters related to yield was adopted by several researchers^45,49,50,72^.

Accurate calculation of daily potential evapotranspiration (ET) is very important for good model performance^40^. DSSAT partitions potential ET into the potential crop transpiration (Ep) and potential soil evaporation (Es)^67^. While actual soil evaporation depends on the potential Es and soil water content, actual crop transpiration is the minimum of potential Ep or root water uptake^73^. The DSSAT model provides two methods for estimating daily potential ET, the FAO-56 Penman–Monteith method^74^ and the Priestley–Taylor^75^ method. The Priestley–Taylor method was used in this study due to unavailability of continuous weather data on wind speed and relative humidity, which are required by the FAO-56 method. Adhikari et al. (2016) used a modified ET method suggested by Thorp et al.^40^, the Priestley–Taylor method gave a good match between the simulated and measured seed cotton yield in this study. The Priestley-Taylor evaporation method was recommended under soil water limited conditions^76^ and it was used in the majority of other DSSAT cotton modeling studies also^77,78^.

### Model performance evaluation statistics

Four statistical indices including the index of agreement (d-index), coefficient of determination (r^2^), percent root mean square error (%RMSE) and percent error (PE) were employed for evaluating the model performance. The d-index is a standard measure of the degree of model simulation error^79^ and it varies between 0 (no agreement) and 1 (perfect agreement) (Eq. 1). The r^2^ measures the fraction of total variance and it varies from 0 to 1^80^ (Eq. 2). A value of 1 represents the perfect co-relation, while the value of 0 indicates that there is no correlation between the measured and simulated values^81^. The %RMSE indicates the average magnitude of the difference between measured and simulated values ^82^ and ranges from 0 to ∞ (Eq. 3). The PE is used to assess systematic over- or under- prediction and varies between − 100 and ∞^83^ (Eq. 4). A value close to 0 indicates a perfect agreement for the %RMSE and PE. The model calibration efforts were continued until the d-index and r^2^ values were higher than 0.80, and %RMSE and PE values were lower than 20.

1 $$ d\hbox{-}index = 1 - \frac{{\mathop \sum \nolimits_{i = 1}^{n} \left( {{\text{Y}}_{{\text{i}}}^{{{\text{sim}}}} - Y_{i}^{mea} } \right)^{2} }}{{\mathop \sum \nolimits_{i = 1}^{n} \left( {\left| {Y_{i}^{sim} - \overline{{Y^{mea} }} } \right| + \left| {Y_{i}^{mea} - \overline{{Y^{mea} }} } \right|} \right)^{2} }} $$

2 $$ r^{2}  = \frac{{\left[ {\sum\limits_{{{\text{i}} = 1}}^{{\text{n}}} {\left( {{\text{Y}}_{{\text{i}}}^{{{\text{mea}}}}  - \overline{{{\text{Y}}^{{{\text{mea}}}} }} } \right)} \left( {{\text{Y}}_{{\text{i}}}^{{{\text{sim}}}}  - \overline{{{\text{Y}}^{{{\text{mea}}}} }} } \right)} \right]^{2} }}{{\left[ {\sum\nolimits_{{i = 1}}^{n} {\left( {{\text{Y}}_{{\text{i}}}^{{{\text{mea}}}}  - \overline{{{\text{Y}}^{{{\text{mea}}}} }} } \right)^{2} } } \right] \times \left[ {\sum\nolimits_{{i = 1}}^{n} {\left( {{\text{Y}}_{{\text{i}}}^{{{\text{sim}}}}  - \overline{{{\text{Y}}^{{{\text{sim}}}} }} } \right)^{2} } } \right]}} $$

3 $$ \% RMSE = \sqrt {\frac{{\mathop \sum \nolimits_{i = 1}^{n} \left( {{\text{Y}}_{{\text{i}}}^{{{\text{sim}}}} - Y_{i}^{mea} } \right)^{2} }}{n}} \times \frac{100}{{ \overline{{Y^{mea} }} }} $$

4 $$ PE = \left[ {\frac{{\mathop \sum \nolimits_{i = 1}^{n} \left( {\overline{{Y^{sim} }} - \overline{{Y^{mea} }} } \right)*\left( {100} \right)}}{{\mathop \sum \nolimits_{i = 1}^{n} \overline{{Y^{mea} }} }}} \right] $$

where $Y_{i}^{sim} , Y_{i}^{mea}$*,*$\overline{{Y^{sim} }}$ *and* $\overline{{Y^{mea} }}$ are the simulated, measured, average simulated and average measured values, respectively.

### Determination of optimum irrigation termination periods for cotton

Using the evaluated CROPGRO-Cotton model, long-term (1978–2019) simulations were run for five deficit/excess irrigation strategies (55% to 115% Evapotranspiration (ET)-replacement in 15% increments) with eight irrigation termination dates with a one-week interval between 15th August and 30th September. The ET-replacement refers to the ET demand which is the accumulation of daily potential plant transpiration plus actual soil evaporation minus infiltration from rainfall (rain—runoff)^84^. The ‘Automatic when required’ irrigation scheduling method was used in this study by keeping the threshold to trigger and stop irrigation at 50% and 100% of available water content, respectively, in the top 30 cm management depth. The estimated amounts of daily irrigation water were considered as the irrigation water requirement for a 100% ET-replacement strategy. Irrigation water requirements for other ET replacement strategies were then estimated by multiplying 100% ET replacement irrigation prescriptions with an appropriate multiplier (e.g., 0.85 for 85% ET replacement scenario). The date-wise estimated irrigation amounts for different ET-replacement strategies were finally input to the model manually. Estimated irrigation applications on all dates after the simulated irrigation termination date were eliminated. Two pre-plant irrigations of 25.4 mm each were applied on 25th and 29th of April in all years for all scenarios to represent a typical practice followed in the study region and adopted in the Halfway experiments^51,61^.

The impacts of environmental conditions during the growing season on cotton IWUE and seed cotton yield under different irrigation termination dates and deficit/excess irrigation strategies were then studied by dividing the simulation period into (i) dry, (ii) normal and (iii) wet years according to the total precipitation received from April 1st to the simulated termination date during the growing season (Supplementary Figure S3). The first year of simulation, 1978, was considered as the model warm-up period and hence it was excluded from the analysis^85^. Years 1985, 1992 and 2015 (extreme wet years) and year 2011 (extreme dry year) were found to be outliers and hence they were also excluded from the analysis. After sorting the remaining 37 years by growing season precipitation, the bottom 12 low-precipitation years were classified as ‘dry’ years and the top 12 high-precipitation years were classified as ‘wet’ years. The remaining 13 years were classified as ‘normal’ years.

Recommendations on ideal irrigation termination periods for cotton production under different ET-replacement strategies were made based on simulated IWUE and yield while keeping in view the HPWD’s annual groundwater extraction limit for irrigation of 460 mm. The IWUE was estimated as the ratio of the difference between irrigated and dryland cotton yield, to the amount of seasonal irrigation water applied^19,20^. As dryland treatment was not included in the field experiment, long-term (1978–2019) simulations were run with the evaluated model for a hypothetical dryland scenario, and the simulated average yield was considered as the dryland yield for estimating IWUE. Based on the simulated yield, the earliest termination date at which the median seed cotton yield reached a peak value or when an increase in median seed cotton yield from that termination date to the next termination date was < 3%, was considered ideal. With reference to IWUE, a termination date corresponding to the maximum simulated IWUE was considered ideal. When the same maximum IWUE was simulated on multiple irrigation termination dates, the earliest irrigation termination date among them was considered ideal. The ideal irrigation termination periods identified based on the simulated median seed cotton yield and simulated median IWUE were then compared, and if they were found to be different, a statistical analysis was carried out using a student’s t-test^86^ to decide an optimum termination period that provides higher seed cotton yield and IWUE. A paired two-tailed t-test was performed (considering the same population twice for two different irrigation termination periods identified based on yield and IWUE) at a significance level of 95% (*P*-value ≤ 0.05) using the T.TEST command in Microsoft Excel.

## Conclusions

The DSSAT CSM CROPGRO-Cotton model was used in this study to assess the effects of irrigation termination dates on IWUE and seed cotton yield under strategic deficit irrigation strategies. Previously evaluated DSSAT CROPGRO-Cotton model^50^ was re-evaluated using in-season crop phenology data and seed cotton yield data collected from a cotton IWUE experiment at Halfway, TX during 2017–2019. The evaluated model performed well in simulating the dates of onset of different cotton phenological stages, canopy height and seed cotton yield. The optimum irrigation termination dates for 55%, 70%, 85%, 100% and 115% ET-replacement strategies were identified as September 12, September 5, September 5, August 29 and August 29, respectively under normal weather conditions. The optimum irrigation termination periods were found to be similar or a week earlier/later than those in normal years in wet/dry years. The 85% ET-replacement strategy along with suggested optimum irrigation termination dates of September 5 in dry and normal years and August 29 in wet years was found to be the most efficient strategy for saving 17 to 41 mm of irrigation water annually. Simulated median irrigation water use was less than the annual groundwater extraction limit (460 mm) specified by the HPWD under all simulated strategies (except for late September irrigation termination dates in case of 115% ET-replacement strategy during dry years) indicating that adoption of appropriate deficit irrigation strategies based on available irrigation capacities could enable producers achieve higher seed cotton yield and IWUE while complying with the HPWD regulations. However, irrigation termination decisions should also consider the differences in water holding capacity of the soil, crop management practices, cultivar characteristics, crop health, and the amount and distribution of late-season rainfall. In addition, extending these simulations to different sites across THP could strengthen the recommendations on irrigation termination decisions. Furthermore, irrigation termination decisions based on modeled soil water content towards the end of the growing season (the time when a producer starts thinking about irrigation termination) could be practically relevant. Our future research efforts will focus on these limitations.

## Supplementary Information

Supplementary Information.
