# Supplementary material for: Assessing the impacts of irrigation termination periods on cotton productivity under strategic deficit irrigation regimes
Source: Sci Rep. 2021 Oct 11;11:20102. doi: 10.1038/s41598-021-99472-w (PMC8505508; doi:10.1038/s41598-021-99472-w)
Supplement: Supplementary file 1 — Supplementary Information. [file 41598_2021_99472_MOESM1_ESM.docx]

**Supplementary Material**

Number of supplementary tables: 5

Number of supplementary figures: 4

**Supplementary Table S1:** DSSAT model parameters adjusted during the model calibration

| **Parameter** | **Description** | **Testing range** | **Calibrated values in Adhikari et al. (2016)** | **Calibrated values in this study** |
| --- | --- | --- | --- | --- |
| **Cultivar Parameters** | | | |  |
| EM-FL | Time between plant emergence and flower appearance (photothermal days) | 34-44 | 42 | 43 |
| FL-SH | Time between first flower and first pod (photothermal days) | 6-12 | 6 | 6 |
| FL-SD | Time between first flower and first seed (photothermal days) | 8-14 | 12 | 10 |
| SD-PM | Time between first seed and physiological maturity (photothermal days) | 38-50 | 42 | 40 |
| FL-LF | Time between first flower and end of leaf expansion (photothermal days) | 55-75 | 55 | 55 |
| LFMAX | Maximum leaf photosynthesis rate at 30 °C, 350 ppm CO_2_, and high light (mg CO_2_ m^−2^ s^−1^) | 0.7-1.4 | 1.1 | 1.05 |
| SLAVR | Maximum leaf photosynthesis rate at 30 °C, 350 ppm CO_2_, and high light (mg CO_2_ m^−2^ s^−1^) | 170-175 | 170 | 170 |
| SIZLF | Maximum size of full leaf (three leaflets) (cm^2^) | 250-320 | 300 | 300 |
| XFRT | Maximum fraction of daily growth that is partitioned to seed + shell | 0.7-0.9 | 0.8 | 0.9 |
| SFDUR | Seed filling duration for pod cohort at standard growth conditions (photothermal days) | 22-35 | 35 | 35 |
| PODUR | Time required for cultivar to reach final pod load under optimal conditions (photothermal days) | 8-14 | 12 | 12 |
| THRSH | Threshing percentage. The maximum ratio of (seed/(seed + shell)) at maturity. | 68-72 | 70 | 71 |
| **Ecotype parameters** | | | |  |
| PL-EM | Time between planting and emergence (photothermal days) | 3-5 | 4 | 4 |
| EM-V1 | Time required from emergence to first true leaf (photothermal days) | 3-5 | 4 | 4 |
| RWDTH | Relative width of the ecotype in comparison to the standard width per node | 0.8-1.0 | 1 | 1 |
| RHGHT | Relative height of the ecotype in comparison to the standard height per node | 0.8-0.95 | 0.9 | 0.80 |
| FL-VS | Time from first flower to last leaf on main stem (photothermal days) | 40-75 | 75 | 75 |
| TRIFL | Rate of appearance of leaves on the mainstem (leaves per photothermal day) | 0.18-0.25 | 0.2 | 0.18 |

**Supplementary Table S2:** Comparison of observed and simulated dates of onset of cotton phenological stages

| **Crop phenological stage** | **Observed days after planting** | | | **Simulated days after planting during calibration** | | | **Simulated days after planting during evaluation** | | |
| --- | --- | --- | --- | --- | --- | --- | --- | --- | --- |
|  | **2017** | **2018** | **2019** | **2017** | **2018** | **2019** | **2017** | **2018** | **2019** |
| Emergence | 5-9 | 5-10 | 5-10 | 6 | 6 | 6 | 6 | 6 | 6 |
| Anthesis | 58-70 | 55-65 | 58-70 | 67 | 63 | 69 | 67 | 64 | 69 |
| Physiological maturity | 145-160 | 135-145 | 135-150 | 157 | 140 | 143 | 156 | 135 | 138 |

**Supplementary Table S3:** Model performance statistics for canopy height simulation

| **Model performance statistics** | | **Calibration** | |  | **Evaluation** | | | |
| --- | --- | --- | --- | --- | --- | --- | --- | --- |
|  |  | **HHH** | **HHH+** |  | **LMH** | **LMH+** | **LMM** | **LMM+** |
|  | **2017** | | | | | | | |
| Index of agreement (d-index) | | 0.99 | 0.99 |  | 0.99 | 0.99 | 0.95 | 0.98 |
| Coefficient of determination (r^2^) | | 0.96 | 0.98 |  | 0.96 | 0.95 | 0.90 | 0.97 |
| Percent root mean square error (RMSE) | | 11.27 | 7.99 |  | 8.88 | 9.65 | 21.43 | 11.78 |
| Average percent error (PE) | | 1.50 | 1.77 |  | -1.98 | -2.48 | -3.71 | 9.09 |
|  | **2018** | | | | | | | |
| Index of agreement (d-index) | | 0.97 | 0.91 |  | 0.93 | 0.96 | 0.90 | 0.98 |
| Coefficient of determination (r^2^) | | 0.91 | 0.90 |  | 0.87 | 0.87 | 0.91 | 0.94 |
| Percent root mean square error (RMSE) | | 11.58 | 20.24 |  | 15.42 | 12.56 | 18.17 | 9.43 |
| Average percent error (PE) | | 2.03 | 11.14 |  | -10.02 | -5.34 | -15.02 | -5.39 |
|  | **2019** | | | | | | | |
| Index of agreement (d-index) | | 0.98 | 0.97 |  | 0.96 | 0.98 | 0.94 | 0.96 |
| Coefficient of determination (r^2^) | | 0.99 | 0.98 |  | 0.98 | 0.98 | 0.99 | 0.97 |
| Percent root mean square error (RMSE) | | 14.10 | 14.63 |  | 13.75 | 9.30 | 15.40 | 13.86 |
| Average percent error (PE) | | 13.04 | 12.54 |  | -8.12 | -4.09 | -12.39 | -6.46 |

**Supplementary Table S4:** Summary of weather parameters during 2017–2019 cotton growing seasons

| **Year** | **Weather parameter^[a]^** | **Month** | | | | | | | **Sum/Average^[b]^** |
| --- | --- | --- | --- | --- | --- | --- | --- | --- | --- |
|  |  | **April** | **May** | **June** | **July** | **August** | **September** | **October** |  |
| **2017** | SRAD (MJ/(m^2^.d)) | 21.9 | 27.5 | 26.7 | 26.9 | 22.5 | 17.7 | 15.7 | 22.8 |
|  | TMAX (˚C) | 23.1 | 27.5 | 32.8 | 32.8 | 29.1 | 27.6 | 23.2 | 28.0 |
|  | TMIN (˚C) | 7.5 | 10.4 | 17.4 | 18.7 | 17.3 | 13.8 | 7.8 | 13.3 |
|  | RAIN (mm) | 68.6 | 34.8 | 83.3 | 73.1 | 132.0 | 93.1 | 20.3 | 505.2 |
|  | WIND (km/h) | 17.6 | 16.6 | 15.2 | 10.5 | 8.8 | 10.8 | 13.7 | 13.3 |
| **2018** | SARD (MJ/(m^2^.d)) | 25.1 | 27.5 | 27.6 | 25.7 | 24.5 | 18.9 | 12.7 | 23.1 |
|  | TMAX (˚C) | 23.2 | 32.6 | 34.8 | 33.5 | 32.5 | 27.5 | 19.7 | 29.1 |
|  | TMIN (˚C) | 3.8 | 14.7 | 18.4 | 19.2 | 18.0 | 14.6 | 8.2 | 13.8 |
|  | RAIN (mm) | 9.2 | 6.6 | 39.4 | 25.9 | 49.7 | 91.7 | 91.4 | 313.9 |
|  | WIND (km/h) | 19.0 | 18.0 | 18.3 | 11.5 | 12.1 | 12.3 | 12.9 | 14.9 |
| **2019** | SARD (MJ/(m^2^.d)) | 22.3 | 22.9 | 27.7 | 27.1 | 24.2 | 19.4 | 15.5 | 22.7 |
|  | TMAX (˚C) | 23.1 | 25.2 | 31.2 | 33.5 | 34.7 | 30.5 | 19.9 | 28.3 |
|  | TMIN (˚C) | 6.0 | 11.6 | 16.4 | 18.7 | 19.3 | 17.3 | 4.8 | 13.4 |
|  | RAIN (mm) | 31.8 | 124.0 | 74.4 | 53.3 | 7.4 | 56.6 | 34.5 | 382.0 |
|  | WIND (km/hr) | 16.1 | 17.1 | 16.3 | 12.4 | 11.7 | 12.8 | 12.9 | 14.2 |

^[a]^ SRAD = Daily total solar radiation, TMAX = Maximum daily temperature, TMIN = Minimum daily temperature, RAIN = Daily total precipitation, WIND = Wind speed

^[b]^ Sum for RAIN and average for other weather parameters

**Supplementary Table S5:** Crop management practices adopted during 2017–2019 cotton growing seasons

| **Management practice** | **2017** | **2018** | **2019** |
| --- | --- | --- | --- |
| **Cultivar** | FiberMax 2011GT | FiberMax 2011GT | FiberMax 2011GT |
| **Planting date** | 15 May | 8 May | 14 May |
| **Seed rate (seed ha^−1^)** | 144903 | 144903 | 119737 |
| **Harvest date** | 15 November | 2 November | 23 October |
| **Irrigation start date** | | | |
| *HHH* | June 20 | June 12 | 2 July^[a]^ |
| *HHH+* | June 20 | June 12 | 2 July |
| *LMH* | July 18 | July 5 | 20 July |
| *LMH+* | July 18 | July 5 | 20 July |
| *LMM* | July 18 | July 5 | 20 July |
| *LMM+* | July 18 | July 5 | 20 July |
| **Irrigation end date** | | | |
| *HHH* | August 29 | August 31 | September 17 |
| *HHH+* | September 15 | September 13 | September 5 |
| *LMH* | August 31 | August 31 | September 17 |
| *LMH+* | September 15 | September 13 | September 5 |
| *LMM* | August 29 | August 31 | September 17 |
| *LMM+* | September 15 | September 13 | September 5 |
| **Seasonal irrigation amount (mm)** | | | |
| *HHH* | 161 | 389 | 362 |
| *HHH+* | 195 | 424 | 385 |
| *LMH* | 105 | 205 | 247 |
| *LMH+* | 138 | 240 | 270 |
| *LMM* | 95 | 151 | 179 |
| *LMM+* | 128 | 187 | 202 |
| **Amount of fertilizer as elemental N, P and Zn (kg ha^−1^)** | | | |
|  | N=78, P=13, Zn=2 | N=103, Zn=1 | N=117, P=66, Zn=1 |

*^[a]^A total of 57 mm of pre-plant irrigation was applied (in multiple applications) for all treatments in the year 2019.*


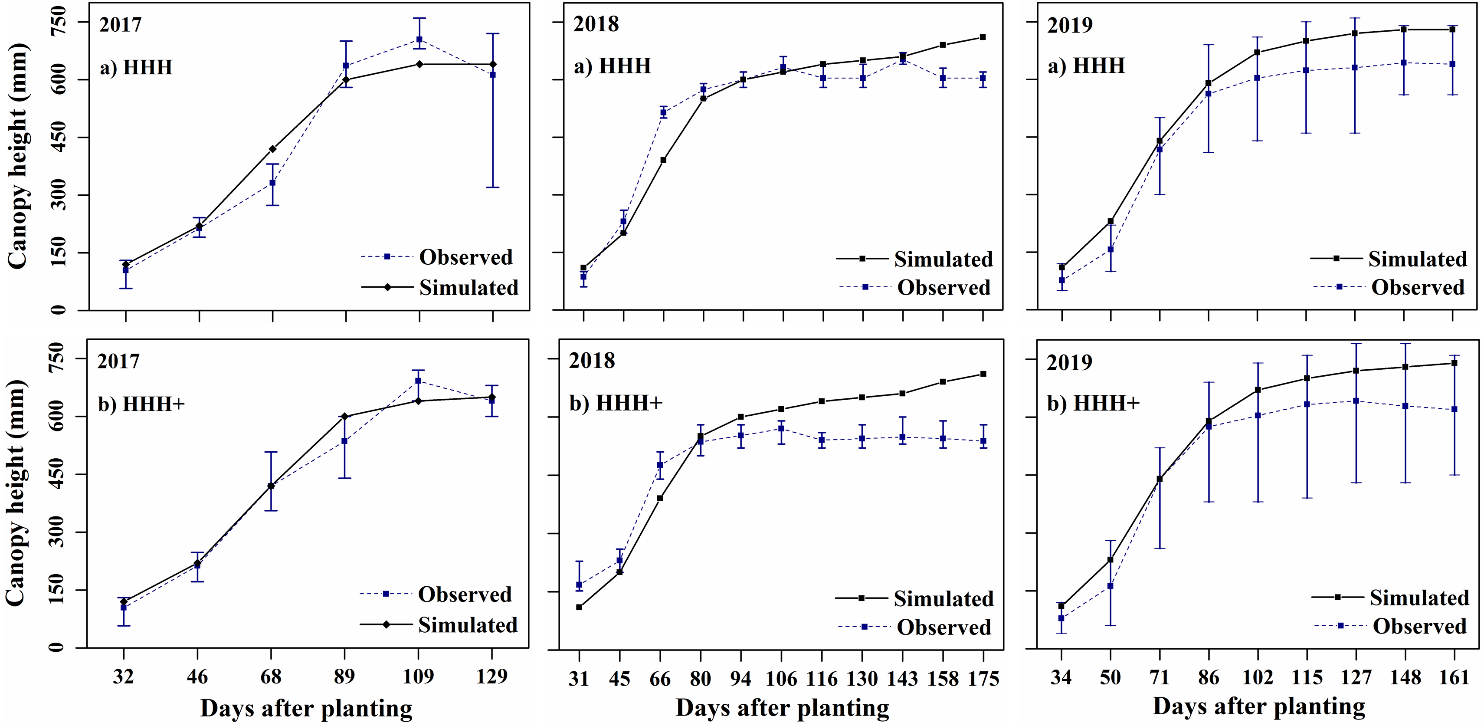


**Supplementary Figure S1:** Comparison of observed and simulated canopy heights during the model calibration for a) HHH, and b) HHH+ treatments. Error bars represent the maximum and minimum observed values (left, center and right columns correspond to 2017, 2018 and 2019, respectively


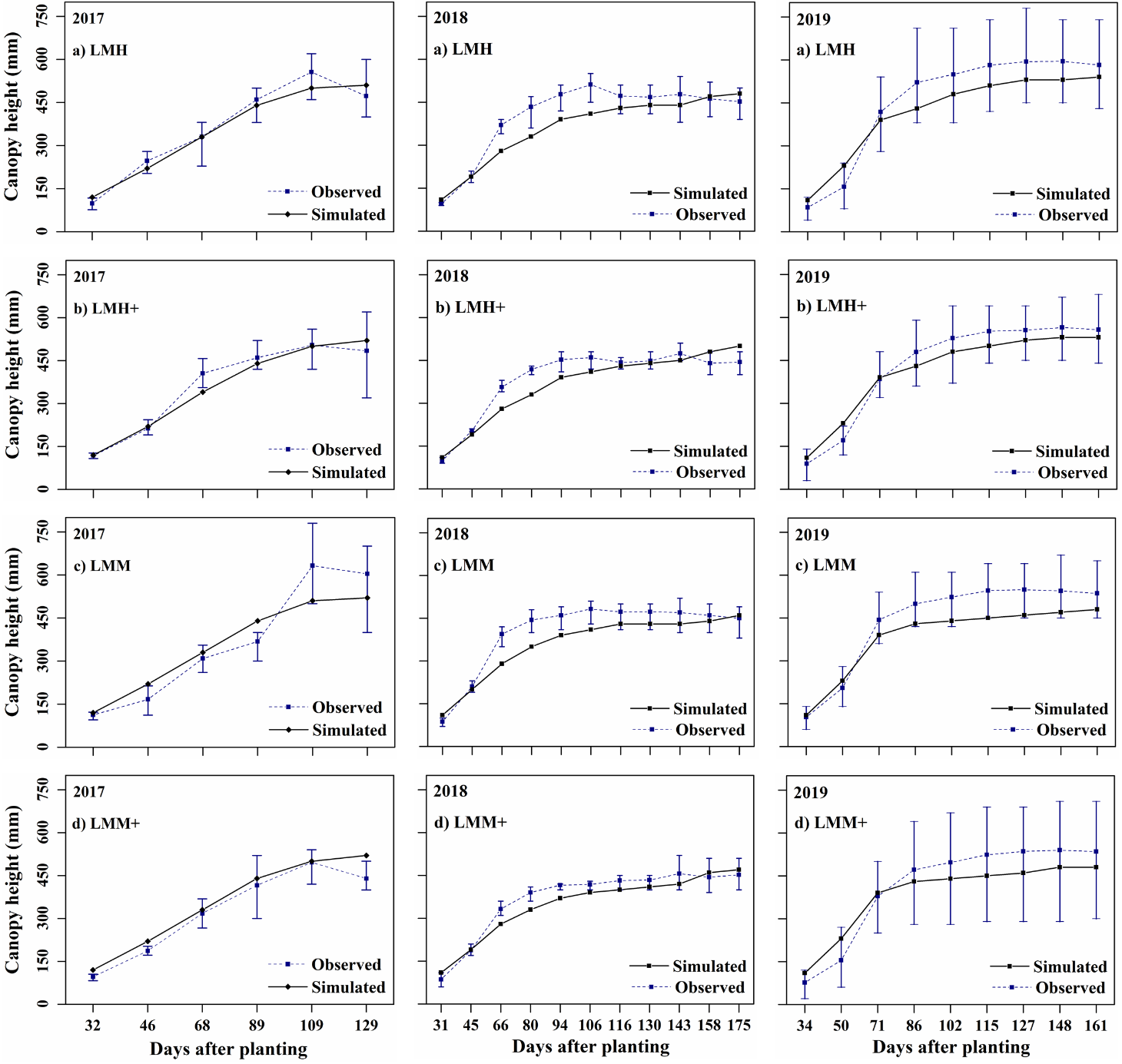


**Supplementary Figure S2:** Comparison of observed and simulated canopy height during the model evaluation for: a) LMH, b) LMH+, c) LMM and d) LMM+ treatments. Error bars represent the maximum and minimum observed values (left, center and right columns correspond to 2017, 2018 and 2019, respectively).


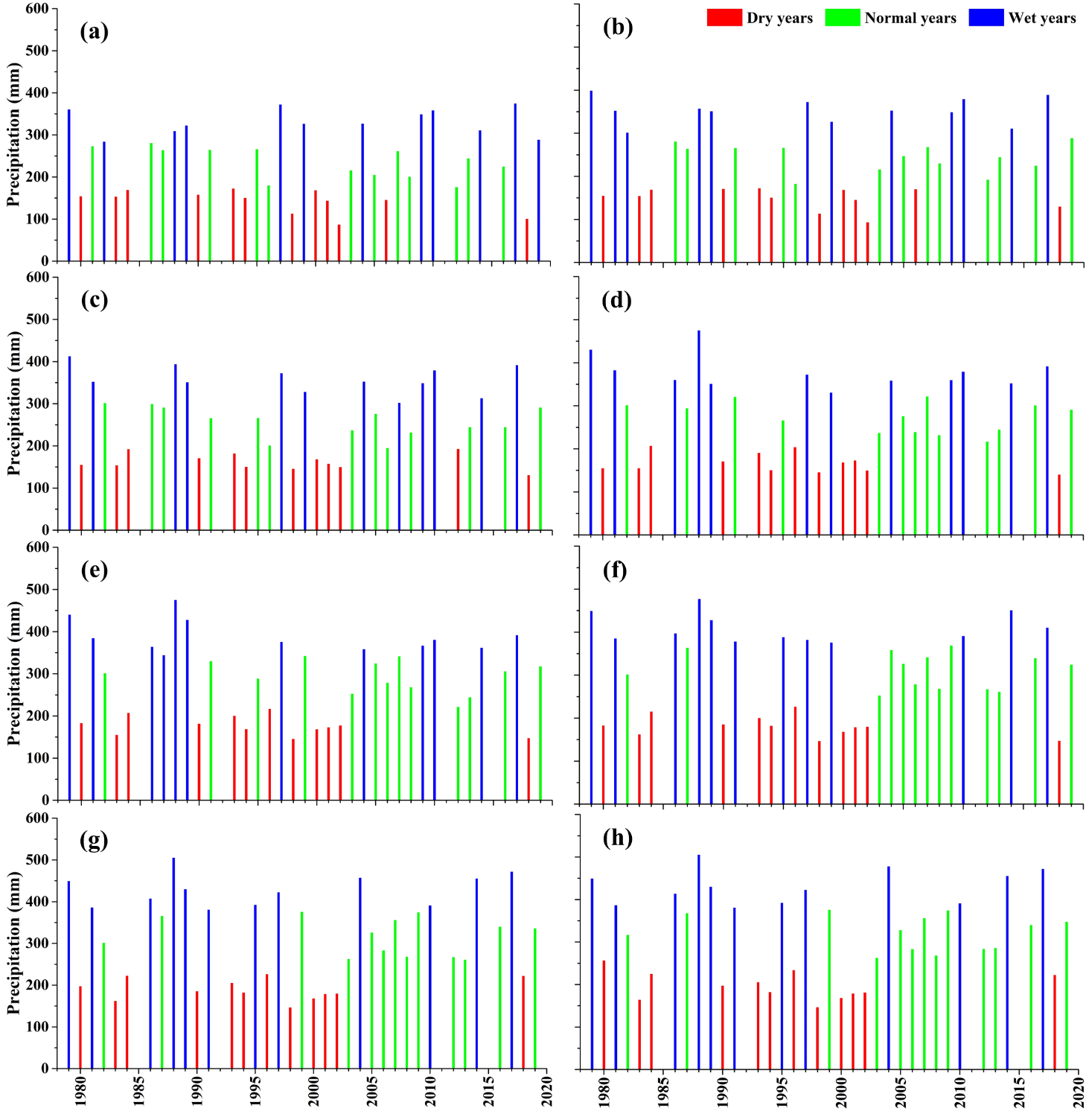


**Supplementary Figure S3:** Classification of the simulation period (1978-2019) into dry, normal and wet years based on the accumulated precipitation during the growing season (from April to the simulated irrigation termination date) at Halfway, TX for different irrigation termination date scenarios: a) August 15, b) August 22, c) August 29, d) September 05, e) September 12, f) September 19, g) September 26, and h) September 30.


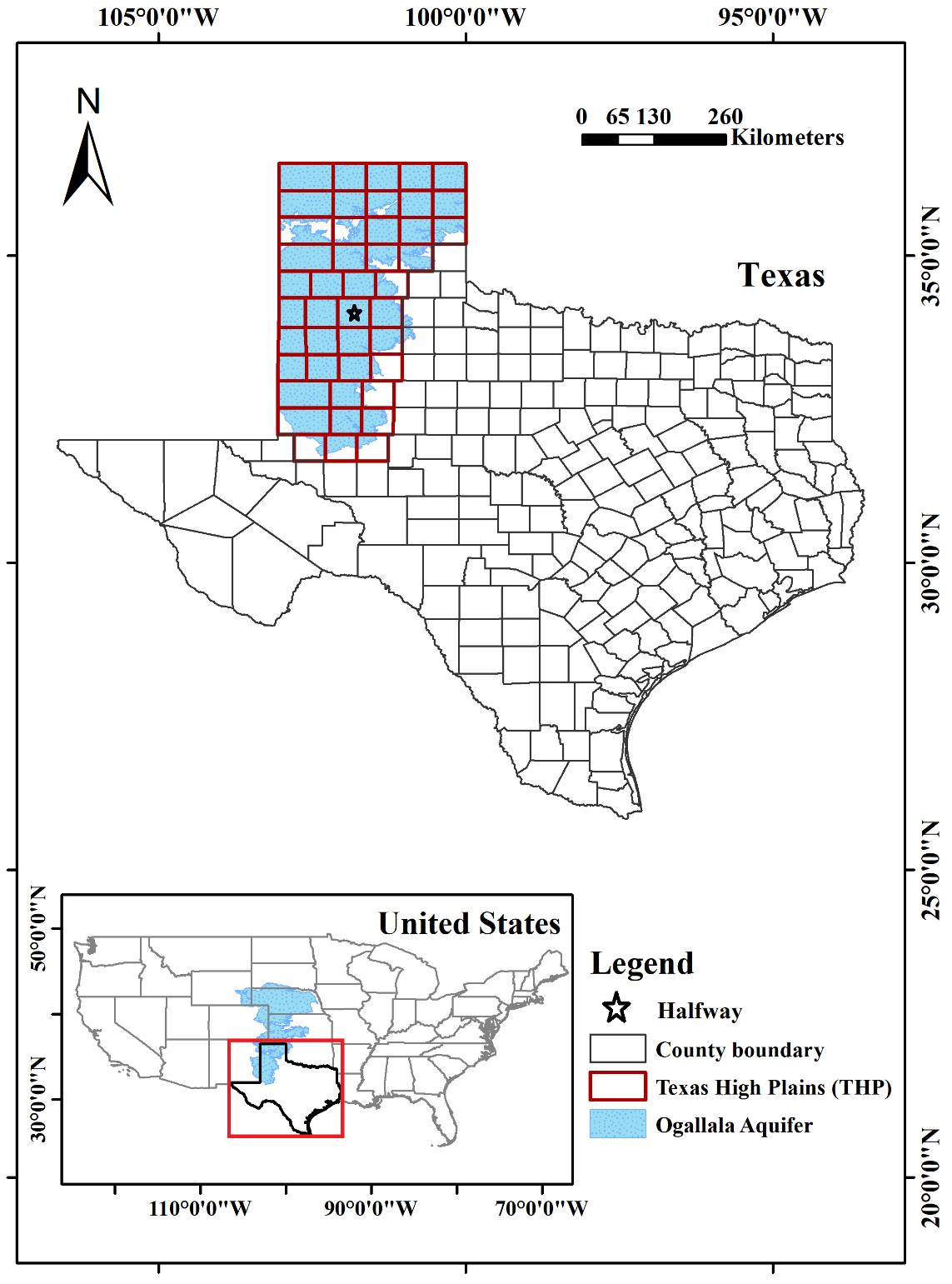


**Supplementary Figure S4:** Location map showing the Texas High Plains (THP) region.
